# Satisfaction of search awareness in trauma radiology: Malignant renal lesion on a trauma thoracolumbar spine CT

**DOI:** 10.1016/j.radcr.2023.04.004

**Published:** 2023-05-12

**Authors:** Alexander M. Satei, Farzad Razi, Huijuan Wang, Serguei Medvedev, Paul J. Arpasi

**Affiliations:** a Trinity Health Oakland Hospital, Pontiac, MI, USA; b Wayne State University School of Medicine, Detroit, MI, USA; c Huron Valley Radiology, Ypsilanti, MI, USA

**Keywords:** Incidental finding, Bosniak classification, Cystic renal lesion, Renal cell carcinoma

## Abstract

Fast-paced trauma imaging can result in misses relating to the nonosseous structures included in the field of view. We report a case of a Bosniak type III renal cyst, later found to be clear cell renal cell carcinoma, incidentally noted on post-traumatic CT of the thoracic and lumbar spine. This case includes a discussion of the circumstances which could result in a radiologist missing this finding, the idea of satisfaction of search, the importance of maintaining a thorough search pattern, and the management and communication of incidental findings.

## Case report

A 77-year-old male presented with severe back pain after a mechanical fall. He denied lower extremity weakness and loss of sensation. Physical examination demonstrated midthoracic point tenderness.

**Fig. 1:**
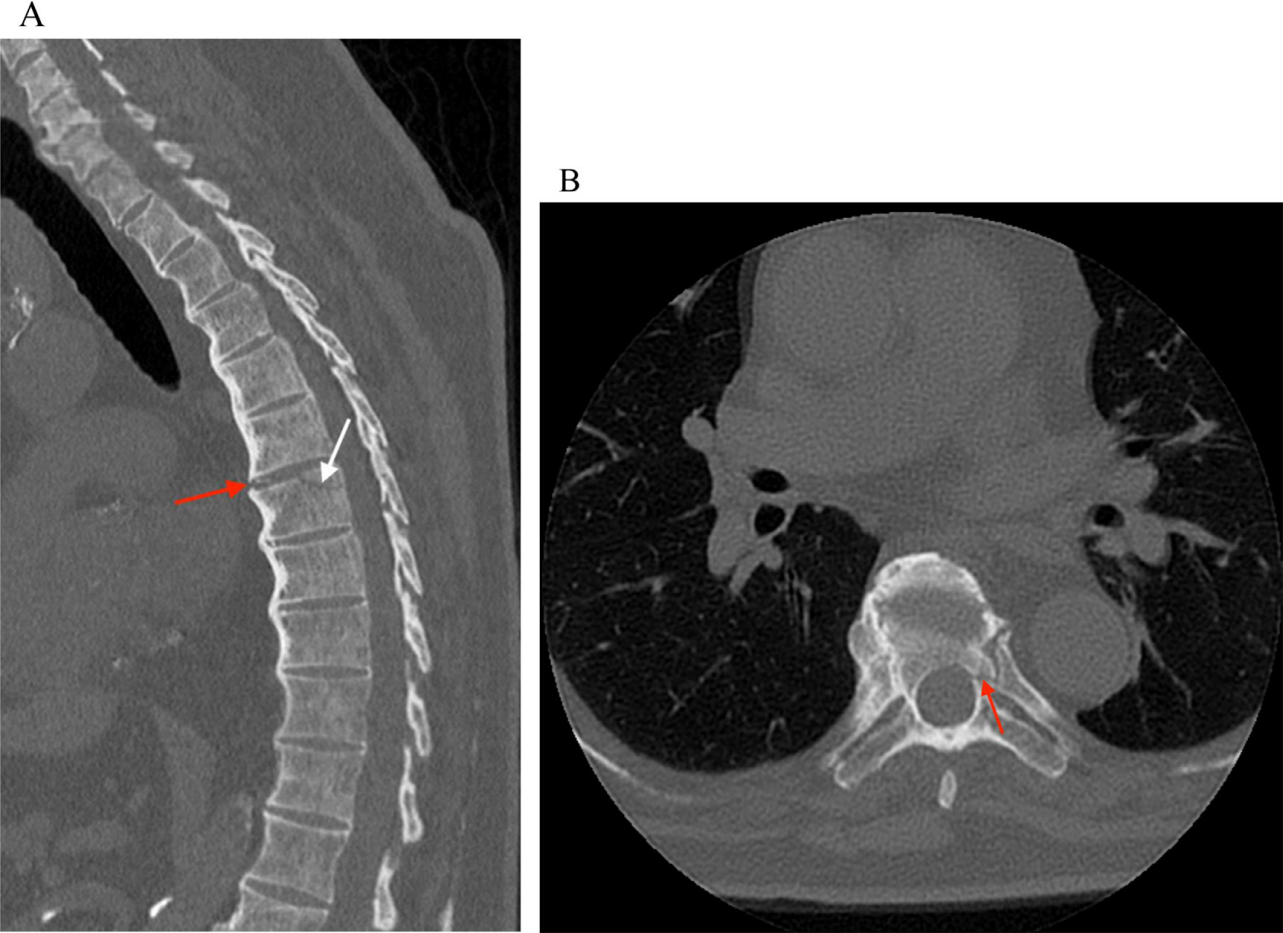
A noncontrast CT image of the thoracic spine. (A) Sagittal CT image demonstrates a nondisplaced oblique fracture of the superior vertebral body of T7 (white arrow). There is widening of the T6-T7 disk space with disruption of the anterior T6-T7 flowing osteophyte (red arrow). (B) Axial CT image demonstrates a nondisplaced linear fracture through the left pedicle of T7 (red arrow).

**Fig. 2:**
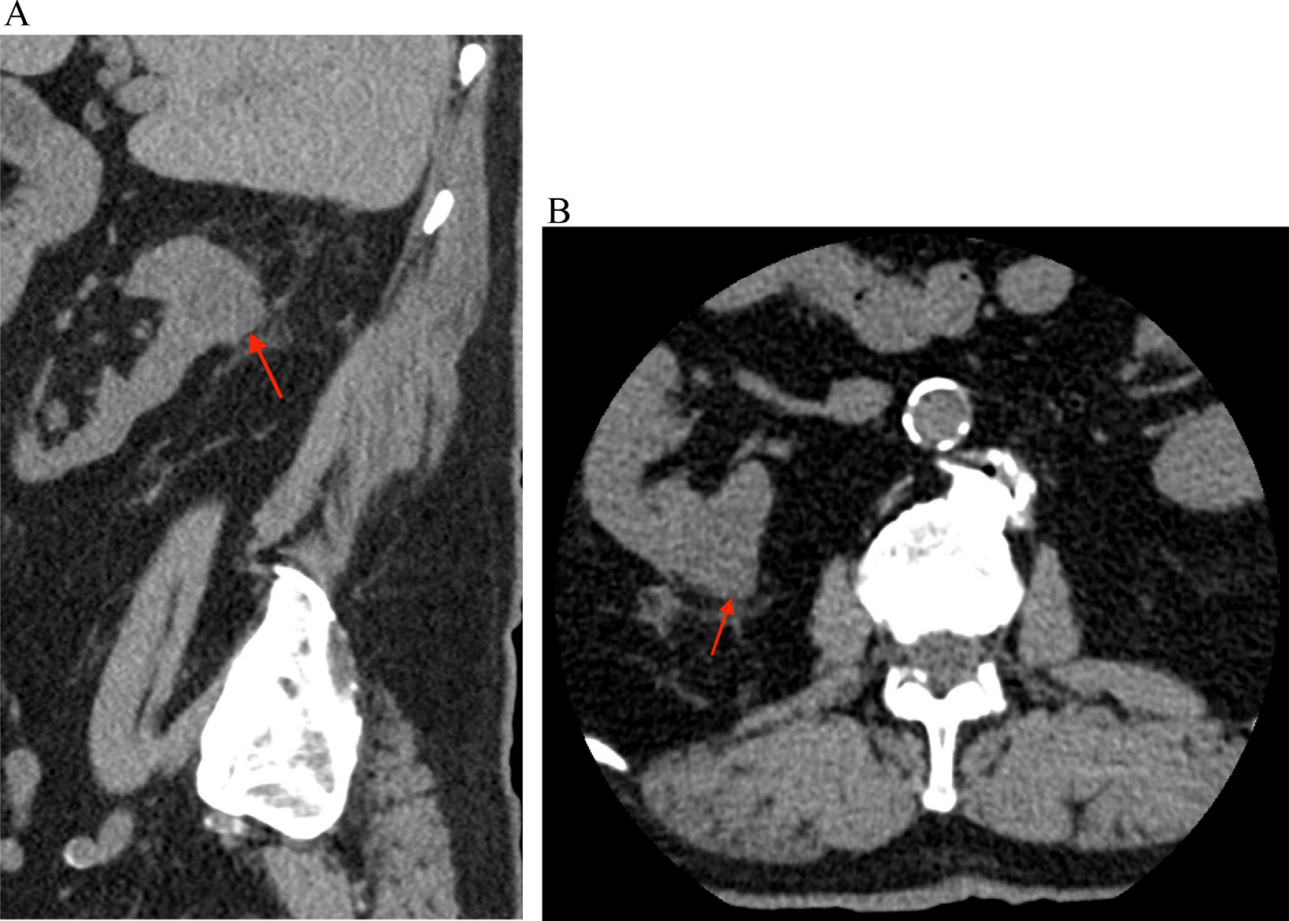
A noncontrast CT image of the lumbar spine. (A) Sagittal and (B) axial CT images reveal an exophytic soft tissue density lesion in the posterior upper pole of the right kidney (red arrow) with surrounding fat stranding.

**Fig. 3:**
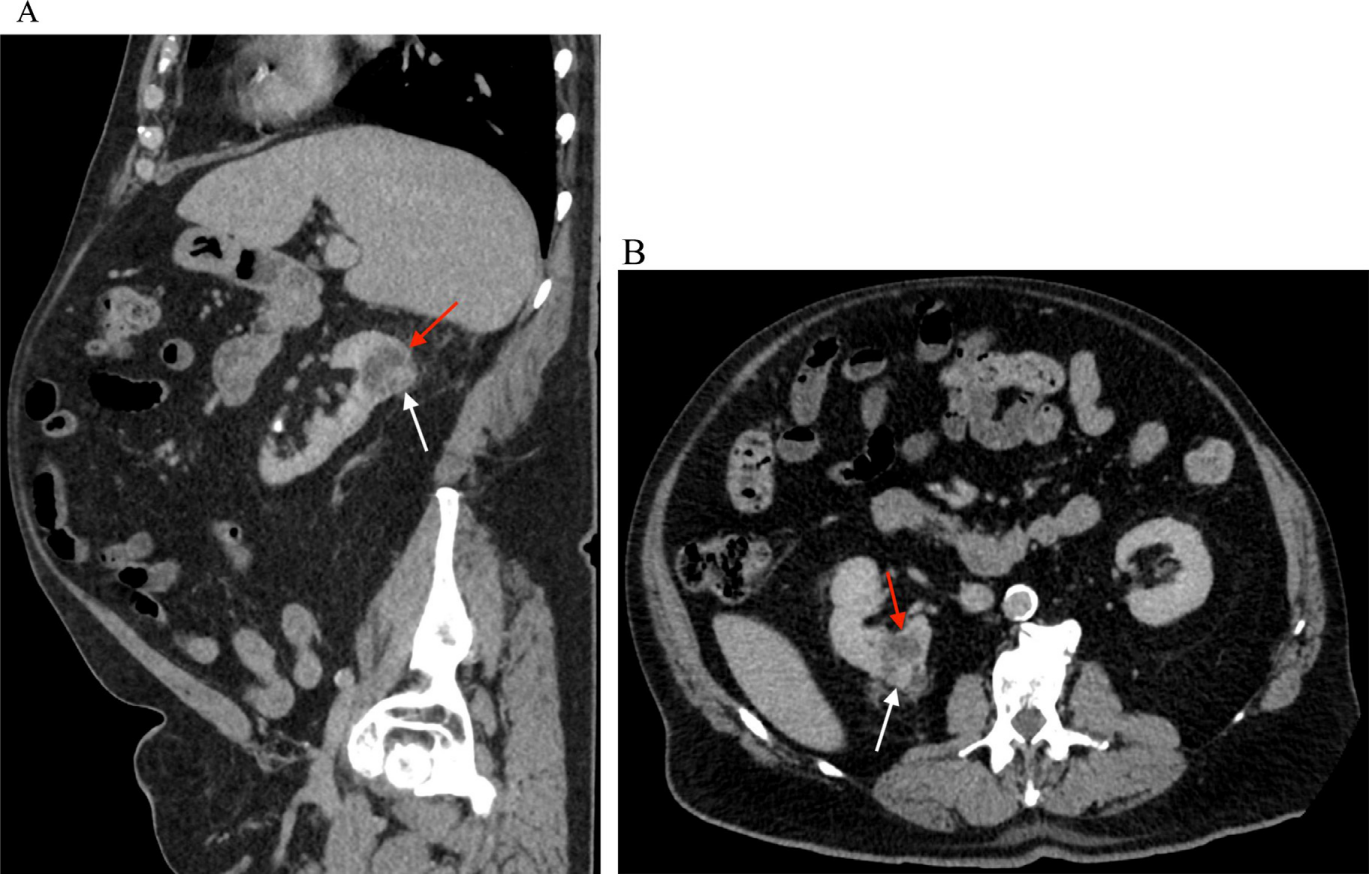
A contrast-enhanced CT of the abdomen and pelvis. (A) Sagittal and (B) axial CT images demonstrate a complex cystic mass in the posterior upper right kidney measuring up to 3.8 × 2.5 × 2.8 cm (red arrow) containing numerous thin and mildly thickened septations and a solid enhancing nodular component along the posterior aspect measuring up to 8 mm in thickness (white arrow).

Computed tomography (CT) of the thoracic and lumbar spine without contrast was performed as part of the patient's initial workup. Initial CT thoracolumbar spine revealed fractures through the superior vertebral body (Fig. 1A) and left pedicle (Fig. 1B) of T7, with widening of the T6-T7 disk space and disruption of the T6-T7 osteophyte (Fig. 1A). Incidentally, an exophytic renal mass was found on the CT of the lumbar spine (Fig. 2). Dedicated nonemergent CT abdomen and pelvis with and without intravenous contrast renal mass protocol was recommended and performed, which revealed a right mid pole renal mass measuring up to 3.8 cm, with numerous internal septations and a 0.8 cm solid nodular component (Fig. 3). This was classified as a Bosniak type III lesion, and subsequent biopsy of this lesion showed clear cell renal cell carcinoma.

## Discussion

The Bosniak classification system stratifies cystic renal masses into 5 categories according to characteristics on CT and/or magnetic resonance imaging (MRI) [1]. The lesion in our report was classified as a Bosniak type III cyst, which is considered to carry an indeterminate probability of malignancy, carrying a 40%-60% risk of malignancy. These cysts typically have thickened irregular or smooth septa with measurable enhancement [2]. In most cases, surgical excision or tissue sampling is recommended.

In our case, the renal cyst was noted on a CT of the thoracic and lumbar spine ordered due to back pain secondary to a mechanical fall. It is therefore classified as an incidental finding and, without the benefit of hindsight, could have been missed if one focused solely on the osseous structures. One of the many pitfalls that could contribute to this is “the satisfaction of search”.

In the context of radiology, a satisfaction of search error occurs when a radiologist prematurely ends their search for other abnormalities after an initial finding, which satisfies the goal of the search, has been identified. Multiple contributory factors play a role in the satisfaction of search. For example, “resource depletion” which refers to reduced attention and memory when processing multiple findings [3]. Another explanation is the idea of “perceptual sets” which refers to the tendency to notice certain details considered more important while disregarding others depending on the radiologist's individual experiences [3]. Reading a study carefully without allowing interference from individual observer factors may prevent satisfaction of search errors. However, it is becoming increasingly difficult for radiologists to find balance between accuracy and speed when their workload continues to increase. In the United States and Canada, growth in CT, MRI, and ultrasound studies between 2012 and 2016 was 1%-5% annually, with growth rates highest among adults 65 years or older [4]. A positive habitual foundation to develop for practicing radiologists and radiology residents is a thorough search pattern. A search pattern should include reviewing all of the images available. This is true and especially important in our case: The renal lesion on axial view is less conspicuous than on the sagittal reformats, thus increasing the likelihood of missing the finding.

One of the secondary effects of a thorough search pattern and resistance to satisfaction of search is an increase in the number of incidental findings. Incidental findings are those that are discovered in the context of imaging ordered for a different reason, such as the renal lesion in our case. A subset of this group, described by the term “incidentaloma,” encompasses masses found unintentionally within the context of the original study ordered [5]. Incidentalomas are identified in about one-third of CT scans performed; however, the rate at which these incidentalomas are malignant ranges from 5% in the brain, parotid, and adrenal glands, to 42% in the breast [6]. The American College of Radiology (ACR) provides recommendations on managing incidental findings depending on the organ involved [7]. In reporting incidental findings, the radiologist must find a balance between the benefits of providing knowledge of a potential adverse diagnosis and the damage caused by overdiagnosing or misdiagnosing them [8]. In our case, the incidental finding was considered significant due to its location and imaging features, and appropriately followed-up with dedicated imaging.

## Patient consent

Informed consent was obtained from the patient for publication of this report and accompanying images.
